# Comparative Transcriptome and DNA Methylation Analysis of Phenotypic Plasticity in the Pacific Abalone (*Haliotis discus hannai*)

**DOI:** 10.3389/fphys.2021.683499

**Published:** 2021-06-29

**Authors:** Zekun Huang, Qizhen Xiao, Feng Yu, Yang Gan, Chengkuan Lu, Wenzhu Peng, Yifang Zhang, Xuan Luo, Nan Chen, Weiwei You, Caihuan Ke

**Affiliations:** ^1^State Key Laboratory of Marine Environmental Science, Xiamen University, Xiamen, China; ^2^College of Ocean and Earth Sciences, Xiamen University, Xiamen, China; ^3^Fujian Key Laboratory of Genetics and Breeding of Marine Organisms, Xiamen University, Xiamen China; ^4^College of Fisheries, Jimei University, Xiamen, China

**Keywords:** transcriptome, DNA methylation, phenotypic plasticity, *Haliotis discus hannai*, climate change, high temperatures

## Abstract

Phenotypic plasticity is an adaptive mechanism used by organisms to cope with environmental fluctuations. Pacific abalone (*Haliotis discus hannai*) are large-scale farmed in the temperate area of northern China and in the warmer waters of southern China. RNA-seq and comparative transcriptomic analysis here were performed to determine if the northern and southern populations have evolved divergent plasticity and if functional differences are associated with protein synthesis and growth-related biological progress. The DNA methylation (5mC) landscape of *H. discus hannai* from the two populations using whole genomic bisulfite sequencing (WGBS), exhibited different epigenetic patterns. The southern population had significant genomic hypo-methylation that may have resulted from long-term acclimation to heat stress. Combining 790 differentially expressed genes (DEGs) and 7635 differentially methylated genes (DMGs), we found that methylation within the gene body might be important in predicting abalone gene expression. Genes related to growth, development, transduction, and apoptosis may be regulated by methylation and could explain the phenotypic divergence of *H. discus hannai*. Our findings not only emphasize the significant roles of adaptive plasticity in the acclimation of *H. discus hannai* to high temperatures but also provide a new understanding of the epigenetic mechanism underlying the phenotypic plasticity in adaptation to climate change for marine organisms.

## Introduction

Phenotypic plasticity involves the ability of organisms to assume different phenotypes, without genotype changes, that facilitates adaptation to environmental variation (Pfennig et al., 2010). Phenotypic plasticity allows rapid adaptation to changing environments and is an important adaptive mechanism for organisms facing the challenges of environmental fluctuations, especially for marine organisms (Li A. et al., 2018; Li L. et al., 2018). Fine-scale adaptive divergence has been observed in populations of marine species facing rapid environmental changes (Place et al., 2012; Kenkel et al., 2013; Li L. et al., 2018). Drastic global climate changes impose a strong selection on marine organisms, especially temperature changes (Sandoval-Castillo et al., 2020; Takeuchi et al., 2020). Populations of some species possess phenotypic plasticity that may buffer the negative impacts of environmental changes. However, there are few published studies on the molecular mechanisms underlying phenotypic plasticity in response to these environmental changes.

The transcriptome provides a useful approach to quantify the plasticity of populations with divergent phenotypic traits that live in heterogeneous environments (Zhou et al., 2012). Alternatively, analysis of DNA methylation shows that it may be capable of regulating gene activity and then shaping fitness-related phenotypic plasticity in species of oysters and fish (Metzger and Schulte, 2016; Wang et al., 2021). Combining transcriptome analysis and DNA methylation may provide a better understanding of the adaptive potential of species (Harrisson et al., 2014).

The Pacific abalone (*Haliotis discus hannai*) is an important aquaculture species in China. It had also been introduced into United States and Chile and accounts for more than 95% of the global abalone production. The commercial culture of *H. discus hannai* in northern China began in the 1980s (Guo et al., 1999). Pacific abalone farming in China has now been extended to subtropical areas (East China Sea), and the abalone population has successfully adapted to the warm water temperatures after 20 years of domestication and selective breeding (Deng et al., 2010; Chen et al., 2017; Chen N. et al., 2018). We previously demonstrated that Pacific abalone from the southern China population had higher thermal tolerance than that from the northern China population. This study was accomplished using the Arrhenius breakpoint temperature (ABT) of cardiac performance (Chen N. et al., 2018), and the results showed phenotypic plasticity divergence in *H. discus hannai*. Large-scale domestication aquaculture activities indicated that Pacific abalone could be a good model for characterizing the mechanisms underlying adaptive phenotypic plasticity in response to elevated temperature.

In the present study, we identified adaptive growth phenotypic divergence in the CNN and CNS populations of *H. discus hannai* associated with long-term cultivation. We also investigated transcriptome differences at gene expression levels using RNA-seq. We examined genomic DNA methylation as a potential epigenetic mechanism underlying phenotypic divergence by whole-genome bisulfite sequencing (WGBS). The association between an altered transcriptome and dynamic methylation increases our understanding of the phenotypic plasticity that helps organisms cope with environmental changes. Our study provides valuable insights into the phenotypic plasticity of *H. discus hannai* and facilitates new understanding of the potential roles of DNA methylation in phenotypic plasticity in marine invertebrates during acclimation to changing environment.

## Materials and Methods

### Growth Performances Differences Between CNS and CNN

The Pacific abalone from southern China (CNS) and northern China (CNN) populations were used here. CNN was the offspring of wild populations from northern China (CNN), while CNS was colonized in warm water after a 20-year period of selection and artificial breeding. The juvenile abalones were collected from CNN (shell length: 19.26 ± 0.49) in Liaoning Province and CNS (shell length: 20.91 ± 0.33) in Fujian province in April 2019. Then abalones from each population were cultivated in 10 suspended sea cages at the Fuda abalone farm in Fujian Province for 1 year. All abalones were fed with *Gracilaria* once every 3 days, with all the residual food particles and fecal debris removed. The sea surface temperature (SST) was recorded every day and then used to calculate the monthly SST. The shell length, and total weight of each individual (randomly selected 90 abalones for each population) were measured and recorded at four intervals including Apr 2019, July 2019, December 2019, and April 2020.

### Sample Collection, RNA/DNA Extraction and Sequencing

Ten adult abalones from CNN (shell length: 63.91 ± 5.46) and CNS (shell length: 65.12 ± 4.56) were chosen, respectively, and cultured in a thermostatic pool at 20 °C. After 7 days of rearing, all of abalones were dissected on Jan 5th, 2018. Since gills of abalone were considered to be sensitive to environmental changes, and available in other mollusks transcriptome study (Chen N. et al., 2018; Zhang X. et al., 2019; Liu et al., 2020). Therefore, we chose the gills as target tissues in our study, and the gills were frozen immediately in liquid nitrogen and then stored at −80°C. Only gills from three samples were randomly selected as biological replicates from each population, which were used for RNA and DNA isolation. Total RNA was isolated using TRIzol Reagent (Life Technologies, Grand Island, NY, United States) followed by DNase I treatment using Qiagen (Valencia, CA, United States) RNeasy Mini columns. The extracted RNA samples were analyzed using a BioAnalyzer (Agilent, CA, United States), and only high-quality RNA samples (RIN > 7) were selected for library preparation. Samples were sequenced independently with the Illumina HiSeq X10 (Illumina, San Diego, CA, United States) by Novogene (Beijing, China).

DNA was isolated using the Universal Genomic DNA Extraction Kit (TaKaRa, DV811A). The purity of DNA was evaluated using ND2000 to ensure that the A260/A280 ratio of DNA was in the range of 1.8 ∼ 2.0. Purified DNA was used for the whole genomic bisulfite sequencing (WGBS) library using a DNA bisulfite conversion kit (TIANGEN, Beijing). The library was then sequenced with an Illumina Hiseq X10 platform (Illumina, San Diego, CA, United States) by Novogene (Beijing, China).

### RNA-Seq Data Analysis

To prepare sequence reads for alignment, sequence adaptors were removed from sequences using Fastp (Chen S. et al., 2018). Sequence reads with a high-quality score were mapped to the Pacific abalone genome (unpublished data) using HISAT2 (Pertea et al., 2016). After the removal of the spike-in controls, an average of 59,760,883 reads were sequenced for each library preparation. There were ∼88.1% sequence reads aligned to the transcriptome. Differential expression analysis was performed using DESeq2 (Love et al., 2014), with a false discovery rate (FDR) ≤ 0.005 and fold change ≥ 2 considered as significantly differentially expressed genes (DEG) between two groups.

### WGBS Data Analysis

Sequencing data were first filtered to remove low-quality reads and aligned to the Pacific abalone reference genome using Bismark2 (Krueger and Andrews, 2011). The methylation level (ML) of the sequence was defined by the following formula: ML(C) = reads(mC)/(reads(mC) + reads(C)). Differentially methylated regions (DMRs) were identified based on the methylation information for each site using DSS software (Park and Wu, 2016) with parameters (smoothing.span = 200, delta = 0, p.threshold = 1e-05, minlen = 50, minCG = 3, dis.merge = 100, pct.sig = 0.5). According to the distribution of DMRs through the genome, we defined the DMRs ambient genes as the DMGs whose gene body region (from TSS to TES) or promoter region (upstream 2 kb from the TSS) had an overlap with the DMRs.

### Functional Enrichment Analysis

Gene Ontology (GO) and Kyoto Encyclopedia of Genes and Genomes (KEGG) pathway enrichment analyses of genes related to DEGs and DMRs were implemented by the clusterProfiler R package (Yu et al., 2012). The significance of the GO terms and pathways was determined by a modified Fisher’s exact test (*p* < 0.05).

### Statistical Analysis

Statistical analyses of study data were performed using the stats R package. All phenotypic traits of abalones from the CNN and CNS populations were shown as mean ± standard deviation (SD). *T*-test analysis was performed on all data to test for significant differences (*p* < 0.05) between groups.

## Results

### Phenotypic Traits Measurements

**FIGURE 1:**
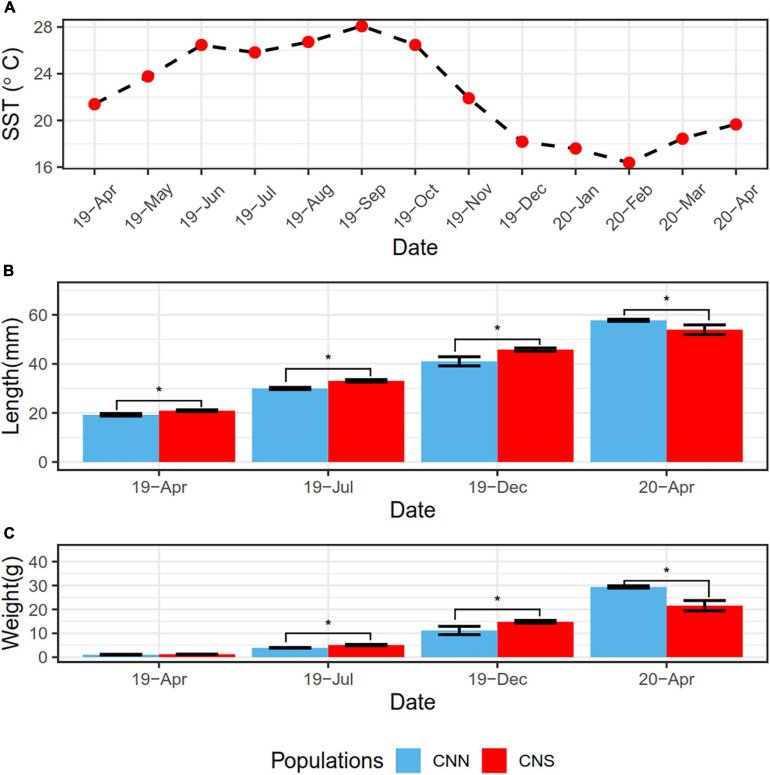
**(A)** Line plot showing the variation of sea surface temperature (SST) from April 2019 to 2020, representing the abalones cultivation environment. **(B)** Bar plot showing the shell length data of *H. discus hannai* from the CNN population and CNS population. **(C)** Bar plot showing the total weight data of *H. discus hannai* from the CNN and CNS populations. The blue bar indicates CNN population and the red bar indicates CNS. *P < 0.05 between two groups.

We studied the physical responses to ambient temperature conditions in the CNN and the CNS populations of *H. discus hannai.* The growth of these abalones was measured from April 2019 to April 2020 (Figure 1). The lengths and weights of abalones from CNS were both significantly greater than those from CNN, probably due to the warm temperature of the sea surface in the summer could be beneficial to the growth of CNS. After overwintering, however, an opposite observation was gained in Apr 2020. The lengths and weights from CNN abalones were both significantly greater than those from CNS, since CNN abalones used to inhabit cold water.

### RNA Sequencing and Mapping

For RNA-seq analysis, a total of 303.68 M reads were generated after critical quality control using fastp, leading to the Q20 varying from 92.23 to 97.93%. An average of 82.97% of the clean data for each sample was mapped to the annotated *H. discus hannai* reference genome (unpublished data), of which 71.18–73.2% had a unique alignment and 5.14–6.1% had multiple alignment positions on the genome (Table 1). After alignment, the abundance of the whole gene was estimated to read count values and normalized by the TPM method.

**TABLE 1:** Summary of the RNA-seq data.

**Samples**	**Clean reads (M)**	**Clean bases (G)**	**Q20 (%)**	**Q20 (%)**	**Mapping rate (%)**	**Unique mapping rate (%)**	**Multiple mapping rate (%)**
CNN_1	45.15	6.77	97.93	93.69	82.29	71.53	6.1
CNN_2	56.99	8.55	97.92	93.71	81.78	71.18	5.45
CNN_3	45.66	6.85	97.49	92.62	83.47	73.1	5.14
CNS_1	44.08	6.61	97.57	92.91	83.63	72.81	5.44
CNS_2	44.11	6.62	97.49	92.70	83.99	73.2	5.32
CNS_3	67.69	10.15	97.29	92.23	82.64	71.67	5.36

### Comparative Transcriptome Analysis in CNN and CNS

**FIGURE 2:**
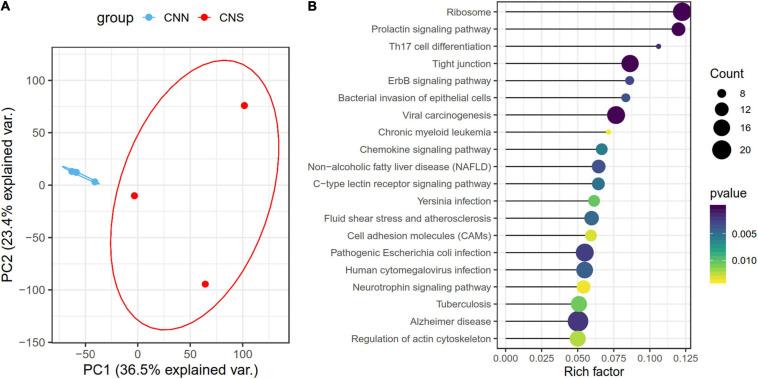
**(A)** Principal component analysis (PCA) of all normalized gene expression levels (TPM) clustered by geographic distribution, demonstrating different transcriptomic patterns of *H. discus hannai* in the two populations. The blue scatters indicate the CNN population and the red one indicates the CNS population. **(B)** Scatter plot showing the top 20 enriched KEGG pathways, resulting from the top 30% contributing genes within the PC1.

Principal component analysis (PCA), based on the normalized gene expression profiles, was conducted. The PCA plot showed that the global patterns of the transcriptomes differed between CNN and CNS populations (Figure 2A). The PC1 explained 36.5% of the variation and revealed a strong relationship between the divergent gene expression pattern of Pacific abalone and geographic distribution. A total of 1,591 genes that belonged to the top 30% contributing genes in the PC1 were identified. KEGG enrichment analysis showed that these genes were significantly enriched in the ErbB signaling pathway, prolactin signaling pathway, regulation of actin cytoskeleton, toll-like receptor signal pathway, and apoptosis (Figure 2B), which are associated with growth and immunity.

**FIGURE 3:**
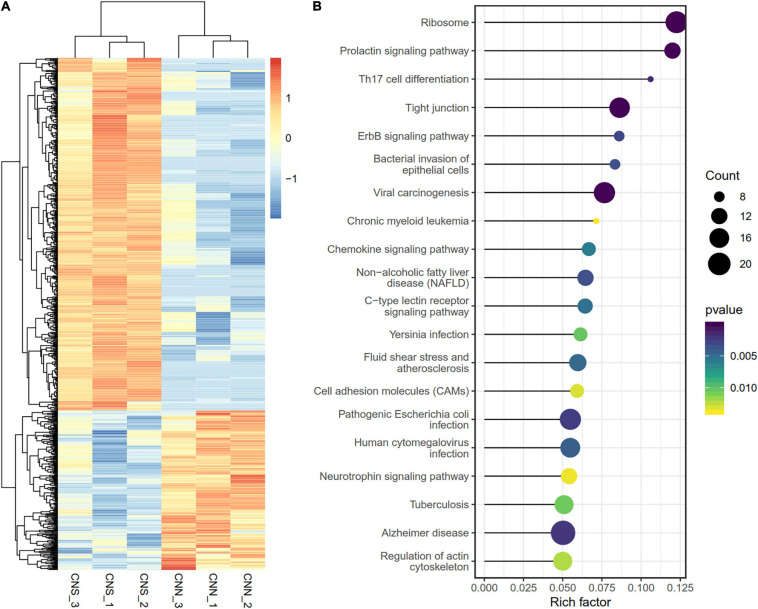
**(A)** Heatmap for the 790 DEGs between the two populations. Rows are genes, and columns are abalone derived from two populations. **(B)** Scatter plot showing the top 20 enriched KEGG pathways, resulting from the DEGs between the two populations.

To determine the transcriptome difference between the two abalone populations, a total of 790 differentially expressed genes (DEGs) (Supplementary Table 1)were identified using DESeq2 R package with | logFC| > 1 and a *p* < 0.05 (Figure 3A). Among these 790 DEGs, most genes (69.6%) were continuously downregulated in the CNN population relative to the CNS population, while only 245 genes were upregulated in CNN. Notably, KEGG enrichment analysis revealed that the top-rank significant pathways also focused on similar biological progresses related to transduction [tight junction, ErbB signaling pathway, cell adhesion molecules (CAMs)], growth (prolactin signaling pathway, regulation of actin cytoskeleton), and neurodevelopment (neurotrophin signaling pathway) (Figure 3B).

### Dynamic Characters of DNA Methylation in CNN and CNS

**FIGURE 4:**
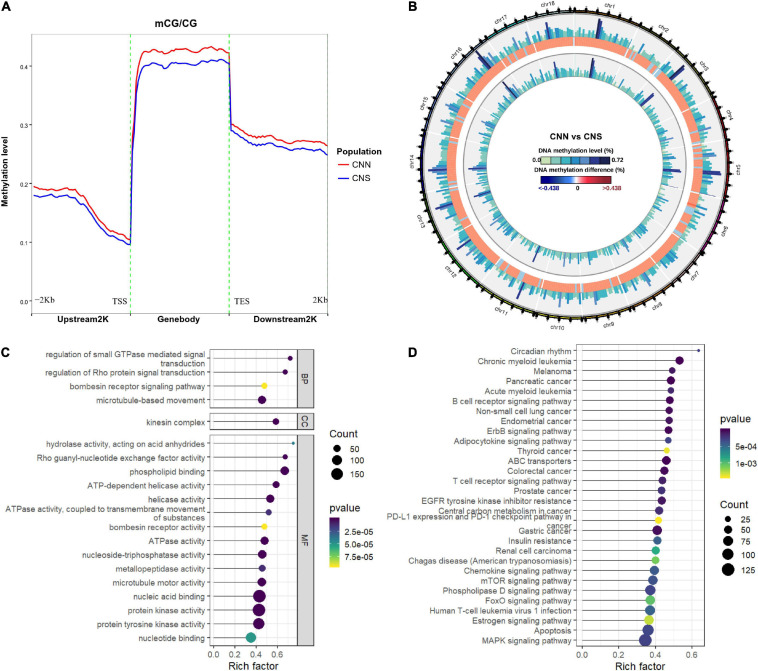
**(A)** Distributions of DNA methylation levels across the whole *H. discus hannai* genomic regions between CNN and CNS populations. **(B)** Circos plot showing the distribution of DNA methylation difference at CG context between CNN and CNS populations across *H. discus hannai* chromosome-scaled genome. The four circles from outer to inner represent chromosomes of *H. discus hannai*, the methylation levels of the CNN population, the differences of methylation level between CNN and CNS population (CNN vs. CNS), and the methylation levels of the CNS population, respectively. **(C)** Scatter plot showing the top 20 enriched GO terms derived from the GO enrichment analysis of DMGs. **(D)** Scatter plot showing the top 20 enriched KEGG pathways derived from KEGG enrichment analysis of DMGs.

We explored the DNA methylation patterns in CNN and CNS abalones using whole genomic bisulfite sequencing (WGBS). These generated a total of 267.98 Gb clean data with an approximately 30× coverage for each sample. The unique mapping efficiencies of these samples varied from 54.56 to 59.06%, and the reads with at least 5 × coverage across the genome accounted for nearly 67.27% of the total reads of each sample. In general, DNA methylation of abalone was primarily found in CG context (83%), and few were found in CHH and CHG contexts (where H is adenine, thymine, or cytosine) (Table 2). The general methylation landscapes of CNN and CNS are shown in Figure 4A, which were calculated independently corresponding to different genome features. Impressively, the average methylation levels at all three contexts were higher in CNN than in CNS (Figure 4A).

**TABLE 2:** Summary of the genome-wide methylation sequencing data.

**Samples**	**Clean bases (G)**	**Q20 (%)**	**Mapping rate (%)**	**BS conversion rate (%)**	**5× coverage (%)**	**mCG (%)**	**mCHG (%)**	**mCHG (%)**
CNN_1	41.29	98.02	59.06	99.912	71.55	84.04	11.78	4.18
CNN_2	42.27	97.47	54.98	99.901	54.79	83.69	12.47	3.84
CNN_3	54.59	97.92	56.56	99.925	70.72	83.71	12.05	4.24
CNS_1	41.11	97.79	57.63	99.942	69.28	87.03	9.44	3.53
CNS_2	42.74	97.6	56.31	99.907	67.62	81.34	13.98	4.68
CNS_3	45.98	97.74	57.15	99.928	69.68	83.89	11.93	4.18

To identify the DNA methylation changes through the whole genome between CNN and CNS, we investigated the differential methylation regions (DMRs) using DSS with a smooth method. A total of 96,565 DMRs (Figure 4B and Supplementary Table 2) were observed and almost all genomic regions were subject to methylation difference. Of these, 50165 CG DMRs, 139 CHG DMRs, and 471 CHH DMRs were significantly hyper-methylated in CNN compared with CNS. Also, 44890 CG DMRs, 432 CHG DMRs, and 468 CHH DMRs were significantly hyper-methylated in CNS. Additionally, the exploration of genomic regions subjected to methylation changes revealed that DMRs were mainly located on repeat region (96565), gene body (16648), and promoter (2011).

To study the potential impact of the DNA methylation differences on the genes, a total of 7635 differentially methylated genes (DMGs) were identified, with the gene body or promoter subject to methylation difference. GO enrichment results showed that most DMGs were significantly enriched in some growth-related terms, such as (GO:0007018) “microtubule-based movement,” (GO:0004672) “protein kinase activity,” and (GO:0035023) “regulation of Rho protein signal transduction” (Figure 4C). Besides, KEGG analysis indicated that major enriched pathways were associated with the ErbB signaling pathway, ABC transporters, MAPK signaling pathway, apoptosis, and FoxO signaling pathway (Figure 4D).

### Relationship Between Altered Transcription and Dynamic DNA Methylation

**FIGURE 5:**
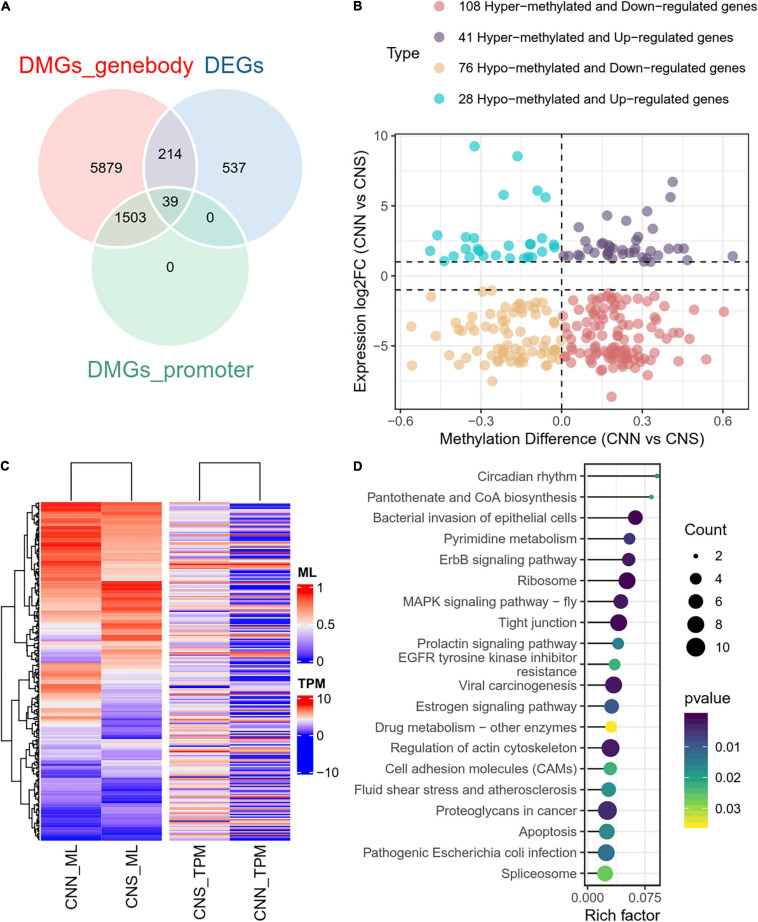
**(A)** Venn plot of overlapped genes of DMGs and DEGs between CNN and CNS populations. **(B)** Scatter plot showing the correlation between the expression log2 fold change and methylation difference in the 253 overlapped genes of DMGs and DEGs between CNN and CNS populations across the *H. discus hannai* chromosome-scaled genome. **(C)** Heatmap showing the methylation levels (ML) and expression profiles (TPM) of the 253 overlapped genes in CNN and CNS populations. **(D)** Scatter plot showing the top 20 enriched KEGG pathways derived from KEGG enrichment analysis of the 253 overlapped genes.

To examine the relationship between DEGs and DMGs in all contexts (CG, CHH, CHG), a total of 253 genes were identified through the examination of overlap of the DEGs and DMGs (Supplementary Table 3), which were derived from gene body and promoter regions (Figure 5A). To further investigate the correlation between expression difference and methylation difference, 108 hyper-methylated and downregulated genes, 28 hypo-methylated and upregulated genes, 41 hyper-methylated and upregulated genes, and 76 hypo-methylated and downregulated genes were identified. These data suggested that relatively more genes had a negative correlation between expression and methylation (Figure 5B). The methylation levels and expression profiles of these overlapped genes in CNN and CNS are shown in a heatmap (Figure 5C). Functional analysis of these DEGs associated with DNA methylation showed that the top-ranking enriched pathways mainly included the ribosome, ErbB signaling pathway, MAPK signaling pathway, regulation of the actin cytoskeleton, and prolactin signaling pathway (Figure 5D), indicating that DNA methylation may play key roles in growth phenotypic divergence between CNN and CNS abalones through transcriptional regulation.

## Discussion

Many studies have demonstrated that long-term environmental changes can lead to dynamics in transcriptome and epigenetic marks. This may result in fine-scale adaptive divergence among populations and help organisms acclimate to the environment (Kenkel and Matz, 2016; Zhou et al., 2018). Our previous study showed that CNS population abalones had greater thermal tolerance abilities than those from the CNN population (Chen N. et al., 2018). This was probably due to the 20-year domestication of CNS in the warm seawater of southern China (Chen et al., 2017). In the present study, a comparison of the average population growth rate showed that the CNS population (1.71 g/month) grew faster than the CNN population (1.26 g/month) in the summer, while the CNN population grew faster (6.01 g/month) than the CNS population (2.24 g/month) in the winter. These data indicated that the two abalone populations exhibited divergent phenotypic plasticity and demonstrated that CNS abalones grow better in warm seawater. The Pacific abalones in China are considered to be a good model to study the impact of divergence in the transcriptome and DNA methylation on phenotypic plasticity in acclimation to a changing environment.

To study the potential molecular mechanisms underlying the divergent phenotypic traits, we performed comparative transcriptome analysis on CNN and CNS abalones and found distinct gene expression patterns in the PCA results. PC1 revealed a strong relationship between the divergent gene expression patterns and geographic distributions. Additional functional analysis of the top 30% contribution to PC1 revealed that most genes were significantly enriched in the pathways related to growth, immunity, and signal transduction. The 790 DEGs also were enriched on similar pathways, such as the ribosome, prolactin signaling pathway, regulation of the actin cytoskeleton, ErbB signaling pathway, toll-like receptor signal pathway, and apoptosis.

Ribosomal proteins are reported to participate in the cellular process of translation and protein synthesis, suggesting vital roles in the growth and development of the organisms (Szakonyi and Byrne, 2011; Lee et al., 2018). Comparative analysis of oysters also indicated that ribosomal proteins may be important in the regulation of growth (Zhang F. et al., 2019). We found 19 proteins that were enriched in the ribosome with significant up-regulation in the CNN population compared to the CNS population. This indicated that conserved ribosomal proteins may play a role in differential efficiency of transcription and protein synthesis. The prolactin signaling pathway has also been shown to play an important role in the growth, development, reproduction, and immune modulation of animals by regulating diverse downstream signaling modules, including JAK/STAT, RAS/RAF/MAPK, and PI3-Kinase/AKT (Radhakrishnan et al., 2012; Bernard et al., 2015; Gorvin, 2015). The functional analysis showed that 12 genes were enriched in this pathway and expressed at high levels in the CNS population. For example, the Forkhead box protein O (FOXO) protein, belonging to the Forkhead family of transcription factors, is important in the regulation of gluconeogenesis and glycogenosis by insulin signaling (Carter and Brunet, 2007). The widespread expression of diverse Fox genes in *Patinopecten yessoensis* suggested that FOX might participate in the regulation of embryo and larval development of mollusks (Wu et al., 2020). Along with the upregulation of FOXO in CNS, the remaining 11 growth-related transcription factors were also found to be expressed at higher levels, including proto-oncogene tyrosine-protein kinase Src (SRC), nuclear factor NF-kappa-B p105 subunit (NFKB1), and signal transducer and activator of transcription (STAT1, STAT5A). The enhanced expression of these growth-related transcription factor genes may contribute to the efficiency of metabolism and protein synthesis, eventually resulting in the growth-related phenotypic divergence between CNN and CNS.

The methylation landscape of *H. discus hannai* showed that DNA methylation is primarily located on the CG, CHH, and CHG components of the genome. Similar results have been documented in the oyster (Wang et al., 2014; Wang et al., 2021). However, the genomic distribution of DNA methylation in *H. discus hannai* is widespread and different from the mosaic methylation patterns observed in *Crassostrea gigas* (Wang et al., 2014). Interestingly, the comparison of DNA methylation levels between CNN and CNS showed that CNN exhibited generally higher DNA methylation levels across different genomic regions. This suggested that CNS abalones may have altered genomic methylation patterns and represent general genomic hypo-methylation in acclimation to the warm temperatures of southern China. Similar methylation patterns were detected in oysters, where intertidal oysters mainly showed genomic hypo-methylation compared to hyper-methylation in subtidal oysters, probably due to their response to heat shock (Wang et al., 2021). These findings suggest that marine mollusks may alter their phenotypic plasticity in DNA methylation in response to warm temperature acclimation. This can be used as a potential biomarker for phenotypic plasticity.

Examination of the widespread DMRs showed that the repeat regions were most subject to epigenetic dynamics, and this may be attributed to the adjacent characteristics between CG and repeats. We found that more DMRs occurred in the gene body than in promoter regions. GO enrichment analysis of DMGs showed that altered methylated genes were significantly overrepresented in microtubules, protein kinase, and nucleotide compound-related GO terms. The KEGG enrichment results indicated that these DMGs were significantly enriched in ABC transporters, MAPK signal pathways, the FoxO signal pathway, and apoptosis, which is similar to the enrichment of DGEs. These results suggest that the CNN and CNS abalone populations exhibit epigenetic divergence. The ATP-binding cassette transporters (ABC transporters) are a classic transport system consisting of membrane proteins that are responsible for ATP-powered translocation (Rees et al., 2009). We found that 54 genes enriched in ABC transporters contained 53 ABC genes that can be classified into six subfamilies (A through G except for E). Among the 53 ABC genes, 26 genes were hyper-methylated in CNS and this may explain the development differences in *H. discus hannai* in response to heat stress. This result suggests that methylation differences on ABC genes can indirectly lead to phenotypic divergence. These findings enhance our understanding of the transport cycle in *H. discus hannai* and suggest that epigenetic regulation may be essential in mediating altered nutrient transport and cell survival under changing environmental conditions.

We studied the relationship between methylation and expression by examining the correlation between the fold changes of DEGs and the methylation difference of DMGs. There was a total of 253 overlapped genes, and all the overlapped genes could be found in the DMGs derived from the altered methylated gene body. This was in contrast to very few derived from promoters and suggested that methylation within the gene body might play an important role in predicting gene expression in abalones. Other animal studies showed that the hyper-methylation of promoters could repress gene expression (Dixon et al., 2018). However, gene body methylation is considered to be one of the essential mechanisms that determine the regulation of gene expression. In this study, we found that relatively more genes showed a negative correlation between expression and methylation. Additionally, KEGG enrichment analysis demonstrated that these 253 specific genes were mainly engaged in the ribosome, prolactin signaling pathway, regulation of actin cytoskeleton, ErbB signaling pathway, and apoptosis.

Seven genes were involved in the apoptotic process, with 5 genes (Lam, Sept4, Lrp11, Ripk1, H-RAS) being hyper-methylated and downregulated, providing evidence that DNA methylation can suppress gene expression. Studies on corals and oysters have illustrated the vital roles of apoptosis in response to high temperature, enhancing survival (Kvitt et al., 2016; Wang et al., 2021). These results indicated that apoptotic processes are regulated by DNA methylation and can contribute to thermal adaptation in abalone (Elmore, 2007). Epigenetic dynamics may be involved in the thermal plasticity of *H. discus hannai*. Incorporating altered expression and dynamic DNA methylation, our results reveal a potential epigenetic mechanism underlying the adaptive phenotypic plasticity of *H. discus hannai*.

## Conclusion

*H. discus hannai* from CNN and CNS populations exhibit divergent phenotypic traits to enable its acclimation to different environments. To study the molecular basis for the phenotypic divergence of *H. discus hannai*, comparative transcriptome and DNA methylation analysis were performed on CNN and CNS. CNN and CNS represented divergent expression patterns with dynamic methylation differences. This suggested that the 20-year domestication of CNS might have accelerated its divergence from CNN in phenotypic traits and expression patterns. Functional analysis of DEGs and DMGs demonstrated that the processes of protein biosynthesis and metabolism are potentially regulated by methylation, which may contribute to the growth-related physiological plasticity of *H. discus hannai*. This study provides insights into the phenotypic plasticity of *H. discus hannai* and the potential roles of DNA methylation in phenotypic plasticity in marine invertebrates acclimating to changing environments.

## Data Availability Statement

The datasets presented in this study can be found in online repositories. The names of the repository/repositories and accession number(s) can be found in the NCBI under the Bioproject accession: PRJNA7177171.

## Ethics Statement

All abalones used for this study were not subject to Institutional Animal Care and Use Committee (IACUC; China) oversight. This study was performed in accordance with the guidelines and regulations for the care and use of animals established by the local institution and government.

## Author Contributions

ZH, WY, and CK conceived and designed the study. ZH, QX, FY, YG, CL, WP, YZ, NC, XL, WY, and CK contributed to participant recruitment, study procedures, and laboratory procedures. ZH and QX performed data analysis. ZH, WY, and CK contributed to the initial manuscript draft. All authors contributed to results interpretation and approved the final version for submission.

## Conflict of Interest

The authors declare that the research was conducted in the absence of any commercial or financial relationships that could be construed as a potential conflict of interest.
